# The Protecting Effects and Mechanisms of Baicalin and Octreotide on Heart Injury in Rats with SAP

**DOI:** 10.1155/2007/19469

**Published:** 2007-12-17

**Authors:** Zhang Xiping, Tian Hua, Chen Hanqing, Chen Li, Wang Zhiwei, Wang Keyi, Yan Wei, Li Yun, Li Qingyu, He Qing, Wang Fei

**Affiliations:** ^1^Department of General Surgery, Hangzhou First People's Hospital, Hangzhou 310006, Zhejiang Province , China; ^2^Department of General Surgery, Second Affiliated Hospital of Zhejiang, University School of Medicine, Hangzhou 310009, Zhejiang Province, China; ^3^Zhejiang University of Traditional Chinese Medical, Hangzhou 310053, Zhejiang Province , China; ^4^Centralab, Hangzhou First People's Hospital, Hangzhou 310006, Zhejiang Province , China; ^5^Manufacturing Laboratory, Hangzhou First People's Hospital, Hangzhou 310006, Zhejiang Province, China

## Abstract

*Purpose.* To observe the protecting effects and mechanisms of Baicalin and Octreotide on heart injury in rats with severe acute pancreatitis (SAP). *Methods.* The SAP rat models were randomly divided into the model group, Baicalin-treated group, Octreotide treated group, and sham operation group. The contents of some inflammatory indexes in blood were determined. The rat mortality, pathological changes of heart, the changes of NF-κB, P-Selectin, Bax, Bcl-2, and Caspase-3 protein expression levels as well as apoptotic index were observed in all groups, respectively, at 3 hours, 6 hours, and 12 hours after operation. *Results.* The survival rate of model group was less than treated groups at 12 hours, difference was significant. The contents of some inflammatory indexes of the treated groups were lower than those of the model group to various degrees at different time points. The pathological myocardial changes under light microscope were milder in treated groups than in model group. The changes of NF-κB, P-Selectin, Bax, Bcl-2, and Caspase-3 protein expression levels in all groups were different. There was only a case of myocardial cell apoptosis in an Octreotide-treated group at 6 hours. *Conclusion.* Baicalin and Octreotide have protecting effects on heart injury of rats with SAP.

## 1. INTRODUCTION

As a common acute abdomen in the clinic, severe acutepancreatitis (SAP) has acute onset and dangerous progression. SAP can easily cause nonpancreas organ injuries, and lead to multiple organ dysfunction (MODS). Its features include dangerous onset, rapid progression and evolvement, multiple complications, great harm, and high mortality [1–4]. The studies on heart injury are relatively few among nonpancreas organ injuries; but the cardiovascular decompensation is one of SAP complications with highest mortality. The clinical examinations often find electrocardiographic abnormality [5, 6], significant change of troponin [7–11], and myocardial zymogram [12, 13]. Its major complicated heart diseases include heart function change, arrhythmia, cardiac shock, toxic myocarditis, pericarditis, myocardial infarction, and so forth. However, there are few drugs to effectively treat such patients.

In “Qing Yi Tang” which is a representative prescription of Chinese medicine for SAP treatment, the enormous clinical practices also suggest its sound therapeutic effects on SAP. Scutellaria baicalensis georgi is a main material in “Qing Yi Tang” while Baicalin (monomer) is its main active constituent. The intravenous administration with very low price can overcome the shortcomings of oral administration of “Qing Yi Tang” including poor absorption and inconvenience. The vitro experiments of Baicalin have proved that it has antibacteria, antivirus, and antiinflammation activities. It also can inhibit platelet aggregation and eliminate oxygen free radicals. In animal experiments, Baicalin with choleretic effectcan can relieve fever, inhibit the thrombin-induced transforming process from fibrin to fibrin, reduce endotoxin generation, and treat and prevent endotoxemia-induced DIC. In addition, the initial metabolite of Baicalin in body is baicalein that can more effectively inhibit pancreatin. All pharmacologic effects can antagonize many processes during SAP onset. Its many effects are similar to those of Somatostatin and its analogues such as Octreotide, but it has a broader application range. It is theoretically feasible to use it for SAP treatment.

As a commonly used drug to treat SAP, Octreotide can alleviate pathological changes of pancreas, reduce ascites, hemodiastase, and lipase, restore pancreatic tissue, inhibit secretion of gastrointestinal hormone and gastric acid, relieve smooth muscle spasm, inhibit local inflammatory reactions, improve pancreas microcirculation, and so forth. Meanwhile, it has defects such as expensive price, short half-life, inconvenient administration, and that it cannot be popularized in remote areas. Therefore, looking for its substitute with low price and good effects is a realistic choice. Baicalin has pharmacologic actions similar to those of Octreotide. It is also cheap and has few side effects and a long half-life. Therefore, it is a promising SAP treating medicine, and has higher research value.

Presently, there has not been any study report on Baicalin treatment of SAP domestically and internationally. This experiment has established the rat SAP model, utilized the tissue microarrays [14], discussed the protecting effects of Baicalin on heart injury in rats with SAP, and compared its effects with those of Octreotide in order to provide the reliable basis for Baicalin treatment of SAP.

## 2. MATERIALS AND METHODS

### 2.1. Materials

Clean grade healthy male Sprague-Dawley (SD) rat 250–300g body weight purchased from the Experimental Animal Center of Medical School, Zhejiang University (Hangzhou, China). Sodium taurocholate and sodium pentobarbital purchased from Sigma-Aldrich (Mo, USA). Octreotide purchased from Novartis (Basel, Switzerland); 5% Baicalin injection (China national invention patent number ZL200310122673.6) prepared by Z. Xiping with 305mmol/L osmotic pressure. The full automatic biochemical analyzer was used to determine the plasma amylase level (U/L). Plasma endotoxin tachypleus amebocyte lysate kit was purchased from Shanghai Yihua Medical Science and Technology Corporation (Institute of Medical Analysis in Shanghai, China). The calculation unit for content is EU/mL. The serum nitrogen monoxidum (NO), malonaldehyde (MDA), superoxide dismutase (SOD) kits were all purchased from Nanjing Jiancheng Bioengineering Research Institute (Nanjing, China). The calculation units for content are, respectively, *μ*mol/L, nmol/mL, and U/mL. The TNF-*α* ELISA kit was purchased from Jingmei Bioengineering Corporation (Shengzhen, China). The calculation unit for content is pg/mL (ng/L). The serum secretory phospholipase A_2_ enzyme Assay ELA kit (PLA_2_) was purchased from R*δ*D system Ins and the calculation unit for content is U/mL. The serum Endothelin-1 ELA kit (ET-1) was purchased from Cayman chemical company (Mich, USA) (catalog number: 583151) and the calculation unit for content is ng/L (pg/mL). The NF-*κ*B, Bax, Bcl-2, and P-Selectinantibody were purchased from Santa Cruz Company (Calif, USA). Caspase-3 antibody was purchased from NeoMarkers Company (Calif, USA), DNA nick in situ end-labeling (TUNEL) kit purchased from Takara BIO INC (Shiga, Japan). The above determinations were all operated according to the instructions of the kits.

### 2.2. Methods

#### 2.2.1. Animal grouping

The improved Aho method was adopted to prepare SAP rat models. The 135 SAP rat models after being prepared were randomly divided into model group, Baicalin-treated group, and Octreotide-treated group with 45 rats in each group; other 45 were selected to be sham operation group, which only received abdomen opening surgery. The above-mentioned groups were then randomly divided into 3 hour, 6 hour, and 12 hour groups with 15 rats in each group, and observation made, respectively, at different points after operation [14–16].

#### 2.2.2. Preparation methods of animal models

The rats were anesthetized by intraperitoneal injection of 2% sodium pentobarbital (0.25mL/100g) after which the rats are laid and fixed, routine shaving, disinfection, and draping are performed, First established the right external jugular vein transfusion passage used the microinfusion pump for continuous transfusion (1mL/h/100g) and then used 3.5% sodium taurocholate to prepare SAP model [14–16].

**Dosage and methods [14–16]:**

1. Baicalin-treated group: The animal experiments of 5% Baicalin injection have been completed including the acute toxicity test and SAP rat treatment by small, middle, and large dose. The large dose can achieve the best therapeutic effect (dose is 10 mg/h/100g) and the dosage referred to the result of the previous preliminary experiment. 10 minutes after successful modeling, Baicalin-treated group was first injected with 5% Baicalin injection 10 mg/100g via external jugular vein passage followed by continuous intravenous administration (10 mg/h/100g) by microinfusion pump; Octreotide-treated group was first injected Octreotide 0.2 ug/100g via external jugular vein passage followed by continuous intravenous transfusion (10 mg/h/100g) by microinfusion pump at a transfusion speed of 0.2 ug/h/100g. All above dosages have been proved as effective dosages in the previous preliminary experiment.
2. Sham operation group and model group: Both of them were injected saline of equivalent volume at the corresponding time points after operation.

Tissue microarrays of heart [17] are prepared as follows.

### 2.3. Observation indexes

1. Survival rate: Examined the rat mortality at 3 hours, 6 hours, and 12 hours after operation and calculated the survival, observed the gross changes of heart.
2. After mercy killing, rats anesthetized by sodium pentobarbital in batches, collected the intestinal samples from heart, fixed them according to the related requirements, and observed the pathological changes of heart after HE staining.
3. The contents of plasma amylase and endotoxin, serum NO, SOD, MDA, TNF-*α*, PLA_2_, and ET-1 were determined via blood sampling from heart.
4. NF-*κ*B, P-Selectin, Caspase-3, Bax, and Bcl-2protein expression: Applied tissue microarrays to prepare heart sections, adopted SP method for immunohistochemical staining, and observed the NF-*κ*B, **P-Selectin, Caspase-3,** Bax, and Bcl-2 protein expression of heart tissue under light microscope, respectively, and carried out the comprehensive assessment according to the positive cell percentage: positive cell count <10% means (—); positive cell count 10–20% means (+); positive cell count 20–50% means (++); positive cell count >50% means (+++).
5. Apoptotic index: Applied the tissue microarrays to prepare the heart microarray sections and adopted DNA nick in situ end-labeling (TUNEL) technology for staining. Observed the intestinal mucosa apoptotic cells and calculated apoptotic index, respectively.

**Statistical methods:**

The values were presented as mean and standard deviation for normal distribution variables or median and quartile range for highly skewed variables. The significance of differences among the four groups was tested using the Kruskal-Wallis test for highly skewed data and analysis of variance (ANOVA) for normal distribution data. Multiple comparisons were subjected to Bonfferoni correction test. The chi-square test was used to evaluate equality of frequencies for discrete variables. Correlations were tested using the Spearman rank correlation coefficients. A *P* value less than or equal to .05 was considered statistical significant, and all statistical analyses were conducted using SPSS version 11.5 for windows.

## 3. RESULTS

**Survival rate:**

The mortalities of model group were, respectively, 0% (0/15), 13.33% (2/15), and 33.33% (5/15) at 3 hours, 6 hours, and 12 hours; all the mortalities of Baicalin-treated group and Octreotide-treated group were 0% at different time points. The whole sham operation group survived at different time points. The survival of model group was 66.67% (10/15) at 12 hours while the survivals of both Baicalin-treated group and Octreotide-treated group were 100% at 12 hours, indicating marked difference (*P* < .05) [14–16].

**Comparison of plasma amylase content in all groups:**

The plasma amylase content of model group and two treated groups significantly exceeded that of sham operation group at different time points (*P* < .001). There was no marked difference between Baicalin-treated group and Octreotide-treated group at different time points (*P* > .05). Although the plasma amylase content of Baicalin-treated group was lower than that of model group at different time points, Baicalin-treated group was significantly less than model group at 3 hours (*P* < .05). There was no marked difference between Baicalin-treated group and model group at 6 hours and 12 hours (*P* > .05). Octreotide-treated group was significantly less than model group at 6 hours (*P* < .05). There was no marked difference between Octreotide-treated group and model group at 3 hours and 12 hours (*P* > .05), see Table 1 [15, 16].

**Comparison of plasma endotoxin contents in all groups:**

The model group and treated groups were significantly higher than the sham operation group at all time points (*P* < .001), no marked difference between the Baicalin-treated group and Octreotide-treated group at 6 hours and 12 hours (*P* > .05). Both the Baicalin-treated group and Octreotide-treated group were significantly lower than the model group at 3 hours (*P* < .001), the Baicalin-treated group was significantly lower than the Octreotide-treated group (*P* < .01). The Baicalin-treated group was significantly lower than the model group at 6 hours (*P* < .05), the Octreotide-treated group was significantly lower than the model group (*P* = .001). The Baicalin-treated group was significantly lower than the model group at 12 hours (*P* < .001), the Octreotide-treated group was significantly lower than the model group (*P* < .01), see Table 1.

**Comparison of serum NO content in all groups:**

Model group, Baicalin-treated group, and Octreotide-treated group all significantly exceeded sham operation group at different time points (*P* < .001). At 3 hours and 12 hours, Baicalin-treated group was significantly less than model group (*P* < .05), Octreotide-treated group was significantly less than model group (*P* < .01). There was no marked difference between Baicalin-treated group and Octreotide-treated group at different time points (*P* > .05), see Table 1.

**Comparison of serum malonaldehyde (MDA) content in all groups:**

The model group, Baicalin-treated group and Octreotide-treated group all significantly exceeded the sham operation group at different time points (*P* < .05). Baicalin-treated group was significantly less than the model group (*P* < .01). Octreotide-treated group was significantly less than the model group at 6 hours and 12 hours (*P* < .05). Baicalin-treated group was significantly less than Octreotide-treated group at 12 hours (*P* < .05), see Table 1.

**Comparison of serum SOD contents in all groups:**

Model group, Baicalin-treated group and Octreotide-treated group were all significantly lower than sham operation group at different time points (*P* < .01), and Octreotide-treated group was significantly higher than model group (*P* < .01). Baicalin-treated group was significantly higher than model group at 6 hours and 12 hours (*P* < .01), and Octreotide-treated group was significantly higher than Baicalin-treated group (*P* < .01), see Table 1.

**Comparison of serum TNF-*α* content in all groups:**

Model group and treated groups significantly exceeded sham operation group at different time points (*P* < .001). There was no marked difference among model group, Baicalin-treated group and Octreotide-treated group at 3 hours and 12 hours (*P* > .05). At 6 hours both Baicalin-treated group and Octreotide-treated group were significantly less than model group (*P* < .001). Octreotide-treated group was significantly less than Baicalin-treated group (*P* < .01), see Table 1.

**Comparison of serum ET-1 contents in all groups:**

Model group was significantly higher than sham operation group at all time points (*P* < .001). At all time points, Baicalin-treated group was significantly lower than model group (*P* < .001), and Octreotide-treated group was significantly lower than model group (*P* < .001). Octreotide-treated group was significantly lower than Baicalin-treated group at 3 hours and 12 hours (*P* < .01). At 3 hours, Baicalin-treated group was significantly higher than sham operation group (*P* < .01), and Octreotide-treated group was not significantly different from sham operation group. At 6 hours, there was no marked difference between Baicalin-treated group or Octreotide-treated group and sham operation group (*P* > .05), or between Baicalin-treated group and Octreotide-treated group (*P* > .05). At 12 hours, Octreotide-treated group and sham operation group had no marked difference (*P* > .05), and Baicalin-treated group was as significantly higher than sham operation group (*P* < .001), see Table 1.

**Comparison of serum PLA_2_ content in all groups:**

Model group and treated groups significantly exceeded sham operation group at different time points (*P* <. 001). At 3 hours, Baicalin-treated group was significantly less than model group (*P* < .01), there was no marked difference between Octreotide-treated group and model group (*P* > .05), Baicalin-treated group was significantly less than Octreotide-treated group (*P* < .01). At 6 hours and 12 hours, Baicalin-treated group was significantly less than model group (*P* < .001), Octreotide-treated group was significantly less than model group (*P* < .001). At 6 hours, there was no marked difference between Baicalin-treated group and Octreotide-treated group (*P* > .05). At 12 hours, Octreotide-treated group was significantly less than Baicalin-treated group (*P* < .001), see Table 2.

## 4. PATHOLOGICAL CHANGES OF MYOCARDIAL TISSUE

**Gross changes:**

Normal-appearing and no marked appearance change were seen in all groups.

**Changes under light microscope:**

The cardiac muscle fiberwas normal in sham operation group, and no cardiac muscle fiber abnormality was found in most other groups. In model group, 2, 1, 1 case of granular or dissolved carcoplasm of rat cardiac muscle fiber occurred, respectively, at 3 hours, 6 hours, and 12 hours. At 6 hours, there were 2 cases of few inflammatory cell infiltrations in myocardial interstitium. At 12 hours, there was 1 case of few inflammatory cell infiltrations in epicardium. In Baicalin-treated group, 2 cases of carcoplasm aggregation occurred at 12 hours; 1, 1, 2 cases of few inflammatory cell infiltrations occurred in rat myocardial interstitium, respectively, at 3 hours, 6 hours, and 12 hours. In Octreotide-treated group, 1 case of carcoplasm aggregation occurred at 12 hours; few inflammatory cell infiltrations occurred in rat myocardial interstitium only once at different time points. The pathological myocardial changes were milder in Baicalin-treated group and Octreotide-treated group than in model group, and Octreotide-treated group had better therapeutic effects.

**Figure 1:**
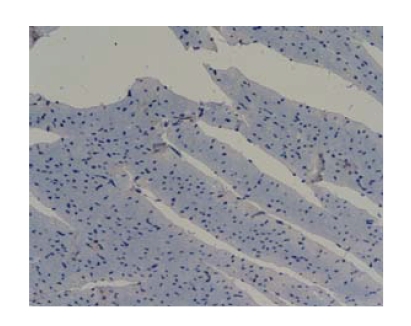
Octreotide-treated group-12 h NF-*κ*B × 200 (negtive expression).

Comparison of NF-*κ*B protein expression levels of myocardial tissue in all groups: The NF-*κ*B protein expression was negative in all groups which showed no marked difference (*P* > .05), see Figure 1.

**Comparison of myocardial tissue P-selectin protein expression levels:**

Myocardial P-selection protein positive staining localized in the cytoplasm and membrane of vascular endothelial cells. The levels of model group at different time points were significantly greater than those of the sham operation group (*P* < .05), and the levels of the Baicalin-treated group at the points of 3 hours and 12 hours were significantly greater than those of the sham operation group (*P* < .05); the levels of the Baicalin-treated group at the points of 3 hours and 6 hours were significantly less than those of the model group (*P* < .01), and the levels of the Octreotide-treated group at different time points were significantly less than those of the model group (*P* < .05); the levels of the Baicalin-treated group at the points of 3 hours and 12 hours were significantly greater than those of the Octreotide-treated group (*P* < .05), see Tables 3 and 4.

**Comparison of Bax protein expression levels of myocardial tissue in all groups:**

**Figure 2:**
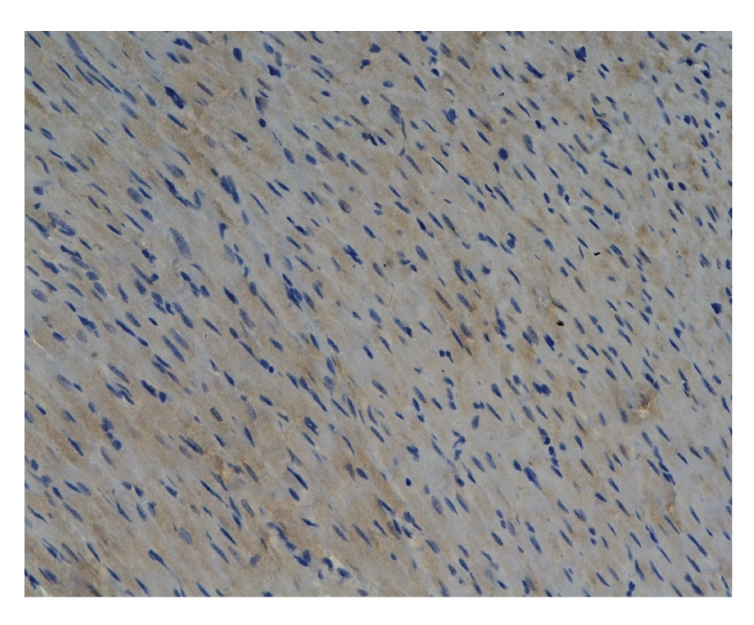
Model group-6h Bax × 200 (positive expression).

**Figure 3:**
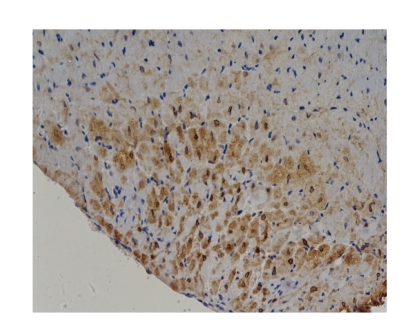
Baicalin-treated group-3h Bax × 200 (positive expression).

**Figure 4:**
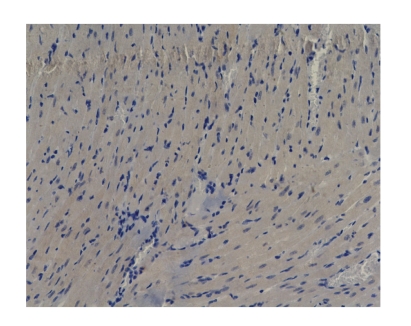
Baicalin-treated group-6h Bax×200 (positive expression).

At 3 hours and 6 hours, model group was significantly higher than sham operation group (*P* < .05). At 6 hours, model group was significantly higher than Octreotide-treated group (*P* < .05). There was no marked difference among other groups (*P* > .05), see Tables 5 and 6, and Figures 2–4.

**Comparison of Bcl-2 protein expression levels of myocardial tissue in all groups:**

**Figure 5:**
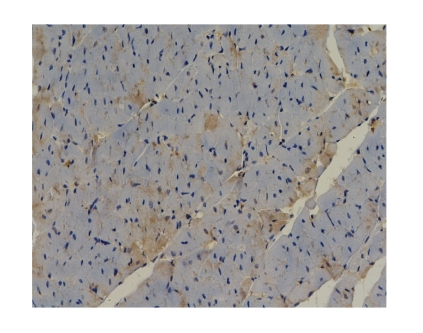
Baicalin-treated group-3h Bcl-2×200 (positive expression).

At 6 hours, Octreotide-treated group was significantly higher than sham operation group (*P* < .01). There was no marked difference among other groups (*P* > .05), see Tables 7 and 8, and Figure 5.

**Comparison of myocardial tissue Caspase-3 protein expression levels:**

Myocardial Caspase-3 protein positive staining mainly localized in the cytoplasm of myocardial cells, and just a little of it positioned in the cytoplasm of vascular endothelial cells. Compared with the sham operation group, there were no significant differences in the model group at different time points and the Octreotide-treated group (*P* > .05) and the levels of the Baicalin-treated group at the points of 6 hours and 12 hours were significantly greater than those of the sham operation group (*P* < .05). Compared with the levels of the model group, there were no significant differences in the Baicalin-treated group and the Octreotide-treated group (*P* > .05), and there were also no significant differences between the Baicalin-treated group and the Octreotide-treated group (*P* > .05), see Tables 9 and 10.

**Comparison of apoptotic index of myocardial tissue in all groups:**

**Figure 6:**
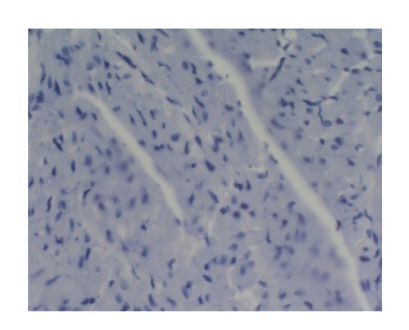
Baicalin-treated group-3h TUNEL×200 (negtive expression).

There was only one case of myocardial cell apoptosis in Octreotide-treated group at 6 hours (apoptotic rate was 2/1000). The others were negative. No marked difference was found among all groups at different time points (*P* > .05), see Figure 6.

**Correlation analysis:**

The amylase was positively correlated with TNF-*α* at 3 hours in model group (*P* < .01). PLA_2_ was positively correlated with TNF-*α* at 12 hours in model group (*P* < .05). Amylase was positively correlated with TNF-*α* at 3 hours in Baicalin-treated group (*P* < .05), and meanwhile TNF-*α* was positively correlated with PLA_2_ (*P* < .01).

## 5. DISCUSSION

Having important impact on SAP onset and progression, inflammatory mediators can cause multiple organ injury. Inflammatory mediators, such as endotoxin, PLA_2_, TNF-*α*, MDA, ET, and NO, have significant roles in SAP pathogenesis. Endotoxin can activate cardiovascular endothelial cell, promote endothelial cell to release a great amount of cytokines, lead to energy metabolism disturbance in myocardial cell, myocardial lipid peroxidation, increase of oxygen free radical, and cause damage of function and structure of cardiovascular endothelial cell and myocardial cell [18–20]. The increase of PLA_2_, which is an important mediator of pancreatic tissue and nonpancreas organ injury after pancreatitis occurs [21, 22], can change ultrastructure of myocardial tissue [23], inhibit calcium pump of myocardial plasma membrane, reduce calcium ion concentration in myocardial cell, and decrease myocardial cell functions [24, 25]. The role of NO in SAP has two aspects [26, 27]. Bulk generation of NO can cause continuous vasodilatation, result in refractory hypotension, lead to myocardial ischemia and anoxemia, or participate in myocardial ischemia reperfusion injury [28]. TNF-*α* can damage heart function by changing intracellular Ca^2+^ balance, lowering myocardial contractility, and inducing NO [29]. Also, MDA, which is an oxygen free radical generated by body through enzymatic system and nonenzymatic system, can indirectly reflect the attack level of oxygen free radical on somatocyte. SOD can eliminate superoxide anion free radical. SOD activity level indirectly reflects body capacity of eliminating oxygen free radical. Free radical can cause glucose and lipid peroxidation, protein denaturation, and enzyme inactivation of cell membrane, break DNA chain in cell, induce apoptosis, and cause heart injury [30, 31]. Endothelin (ET) can lead to myocardial ischemia necrosis [32, 33]and damage heart structure and function through its vascular contractile effect [34, 35]. It also can cause heart ischemia anoxemia, or even thrombosis [36].

P-Selectin is a member of the family of cell adhesion molecule and is expressed in most architectonic blood vessels of the normal human body. However, the content is very low and the expression can be significantly increased when in acute inflammatory [37, 38]. It is also an important indicator of inflammation [37, 39].

This study found that, compared with the sham operation group, P-Selectin protein expression of the model group was significantly upregulated, and it can be further intensified as the disease progressed. It is shown that P-Selectin involved in the pathological process during the SAP heart damage period. However, P-Selectin protein expression in the Baicalin and Octreotide-treated groups was decreased to varying degrees; and at the same time, the heart pathological lesion was improved. All that shows these two kinds of medicine have some certain therapeutic effects.

This experiment has fully observed the influence of Baicalin and Octreotide on inflammatory mediators of SAP rats, and discussed their heart protecting effects. Study results showed that both amylase content in plasma and content of endotoxin, PLA_2_, NO, TNF-*α*, MDA, and ET-1 in serum were lower in Baicalin-treated group and Octreotide-treated group than in model group, while their SOD contents were higher than that of model group. The content of endotoxin, PLA_2_ and ET-1 dropped significantly in Baicalin-treated group at all time points, and was close to that in Octreotide-treated group. Compared with model group, the serum NO at 3 hours and 12 hours (*P* < .05), serum TNF-*α* at 6 hours (*P* < .01), and plasma amylase at 3 hours (*P* < .05) dropped significantly in Baicalin-treated group. Comparison of myocardial tissue P-Selectin protein expression levels shows that the levels of the Baicalin-treated group at the points of 3 hours and 6 hours were significantly less than those of the model group (*P* < .01), and the levels of the Octreotide-treated group at the points of 3 hours, 6 hours, and 12 hours were significantly less than those of the model group (*P* < .05). This experiment showed that the Bax protein expression level of myocardial tissue at 3 hours and 6 hours was higher in model group than in sham operation group (*P* < .05), and model group was significantly higher than Octreotide-treated group at 6 hours (*P <* .05). The Bcl-2 protein expression level of myocardial tissue at 6 hours was significantly higher in Octreotide-treated group than in sham operation group (*P* < .01). The levels of myocardial tissue Caspase-3 protein expression in the Baicalin-treated group at the points of 6 hours and 12 hours were significantly greater than those of the sham operation group (*P* < .05). There was only a case of myocardial cell apoptosis in Octreotide-treated group at 6 hours, and other groups were negative. The NF-*κ*B protein expression of myocardial tissue was negative in all groups. In addition, the NF-*κ*B protein expression was negative in all groups at different time points, indicating no NF-*κ*B expression in heart. Therefore, NF-*κ*B cannot directly act on heart. Bax and Bcl-2, respectively, can induce and inhibit apoptosis.

Caspase-3 is one of the important proteases which can induce apoptosis and is also the final effect factor of the Caspase cascade effect which is involved in apoptosis, and moreover it is at the core position in the process of cutting protease cascade. Caspase-3 is a marker of apoptosis and it is also the performer of apoptosis. It can destroy a variety of protease complex in cells with the digestive way, activate intranuclear nuclease to cause the DNA schizolysis form the DNA fragments, undermine cell calcium pump function, lead to the situation of intracellular calcium overload, and so on [40, 41]. Inhibiting Caspase-3 activity can reduce the occurrence possibility of apoptosis [42].

In this study, we found that the levels of myocardial tissue Caspase-3 protein expression in the Baicalin-treated group were significantly greater than those of the sham operation group. Compared with the model group, there were no significant differences in the Baicalin-treated group and the Octreotide-treated group, and there was no significant difference between the Baicalin-treated group and the Octreotide-treated group. It is shown that the role of Baicalin and Octreotide inducing myocardial apoptosis may be irrelevant to Caspase-3 in SAP. The results of this experiment showed apoptosis had limited and harmful effects in heart injury.

The experiment showed us that Baicalin and Octreotide can effectively lower the level of inflammatory mediators, and have protecting effects on hearts of SAP rats. In addition, this experiment has applied tissue microarrays, whose advantages include high throughput, multiple samples, cost and time saving, low error, convenience for experimental control design, capability to combine other biotechnologies, and extensive applications to greatly lower study cost, improve the efficiency of pathohistological study, and achieve satisfactory results. We have not seen this method applied in pancreas pathological study till now. This article has reported its application for the first time. We believe it is worth popularization.

## Figures and Tables

**Table 1:** Comparison of different indexes level in blood ($M\left(Q_{R}\right)$)

Indexes	Time point	Sham operation group	Model group	Baicalin-treated group	Octreotide-treated group
Amylase (U/L)	3h	1582.00(284.00)	5303.00(1373.00)	4342.00(1496.00)	5419.00(1670.00)
	6h	1769.00(362.00)	6276.00(1029.00)	5130.00(1591.00)	5058.00(1314.00)
	12h	1618.00(302.00)	7537.50 (2933.50)	5571.00(2307.00)	6531.00(2280.00)
Endotoxin (pg/mL)	3h	0.016(0.005)	0.053(0.029)	0.027(0.005)	0.033(0.006)
	6h	0.016(0.010)	0.059(0.037)	0.039(0.019)	0.031(0.010)
	12h	0.014(0.015)	0.060(0.022)	0.034(0.015)	0.042(0.014)
NO (pg/mL)	3h	7.500(5.00)	65.00(7.50)	57.50(22.50)	52.50(15.00)
	6h	7.500(5.00)	62.50(38.75)	47.50(37.50)	57.50(15.00)
	12h	10.00(5.00)	74.10(26.15)	57.50(27.50)	45.00(12.50)
MDA (nmol/mL)	3h	9.90(9.90)	36.30(13.40)	21.90(13.45)	29.60(18.60)
	6h	16.50(13.20)	39.70(9.90)	23.80(14.60)	33.00(9.90)
	12h	16.50(13.20)	54.35(19.00)	36.00(11.60)	40.30(16.80)
TNF-*α* (pg/mL)	3h	3.90(3.20)	41.44(37.72)	44.93(45.8420)	39.30(30.60)
	6h	4.00(1.70)	92.15(23.12)	65.10(27.51)	47.60(16.50)
	12h	5.30(3.00)	65.02(26.81)	47.65(25.52)	54.50(41.40)
ET-1 (pg/mL)	3h	15.293(4.231)	24.745(1.011)	19.635(6.065)	16.827(3.775)
	6h	16.275(3.180)	25.625(7.973)	16.226(3.174)	14.855(5.747)
	12h	14.173(2.556)	24.725(3.759)	18.625(5.780)	15.185(1.761)

**Table 2:** Comparison of serum PLA_2_ content in all groups ($\bar{X}\pm S$).

Groups	3 hours	6 hours	12 hours
Sham operation group	14.6170±3.0160	17.4860±3.8205	19.0187±5.0660
Model group	76.0941±16.7036	101.4595±14.6662	105.3285±18.1015
Baicalin-treated group	56.2481±22.4331	67.9088±20.6117	66.8585±22.0971
Octreotide-treated group	74.3729±19.9352	63.1289±26.3061	53.6335±12.2774

**Table 3:** Changes of P-selectin protein expression in all groups.

Groups	Cases	Pathologic grade			
		−	+	++	+++
Sham operation group (3h)	15	15	—	—	—
Sham operation group (6h)	15	15	—	—	—
Sham operation group (12h)	15	15	—	—	—
Model group (3h)	15	3	5	7	—
Model group (6h)	13	5	7	1	—
Model group (12h)	10	7	2	1	—
Baicalin-treated group (3h)	15	10	5	—	—
Baicalin-treated group (6h)	15	13	2	—	—
Baicalin-treated group (12h)	15	11	4	—	—
Octreotide-treated group (3h)	15	15	—	—	—
Octreotide-treated group (6h)	15	15	—	—	—
Octreotide-treated group (12h)	15	15	—	—	—

**Table 4:** Comparison of P-Selectin protein expression in all groups ($M\left(Q_{R}\right)$).

Groups	3 hours	6 hours	12 hours
Sham operation group	0.00(0.00)	0.00(0.00)	0.00(0.00)
Model group	1.00(1.00)	1.00(1.00)	0.00(1.00)
Baicalin-treated group	0.00(1.00)	0.00(0.00)	0.00(1.00)
Octreotide-treated group	0.00(0.00)	0.00(0.00)	0.00(0.00)

**Table 5:** Changes of Bax protein expression in all groups.

Groups	Cases	Pathologic grade			
		−	+	++	+++
Sham operation group (3h)	15	15	—	—	—
Sham operation group (6h)	15	15	—	—	—
Sham operation group (12h)	15	15	—	—	—
Model group (3h)	15	10	1	4	—
Model group (6h)	13	8	2	3	—
Model group (12h)	10	10	—	—	—
Baicalin-treated group (3h)	15	14	—	1	—
Baicalin-treated group (6h)	15	14	—	—	1
Baicalin-treated group (12h)	15	15	—	—	—
Octreotide-treated group (3h)	15	14	1	—	—
Octreotide-treated group (6h)	15	14	1	—	—
Octreotide-treated group (12h)	15	15	—	—	—

**Table 6:** Comparison of Bax protein expression in all groups ($M\left(Q_{R}\right)$).

groups	3 hours	6 hours	12 hours
Sham operation group	0.00(0.00)	0.00(0.00)	0.00(0.00)
Model group	0.00(2.00)	0.00(2.00)	0.00(0.00)
Baicalin-treated group	0.00(0.00)	0.00(0.00)	0.00(0.00)
Octreotide-treated group	0.00(0.00)	0.00(0.00)	0.00(0.00)

**Table 7:** Changes of Bcl-2 protein expression in all groups.

Groups	Cases	Pathologic grade			
		−	+	++	+++
Sham operation group (3h)	15	15	—	—	—
Sham operation group (6h)	15	15	—	—	—
Sham operation group (12h)	15	15	—	—	—
Model group (3h)	15	13	1	1	—
Model group (6h)	13	12	1	2	—
Model group (12h)	10	10	—	—	—
Baicalin-treated group (3h)	15	14	—	1	—
Baicalin-treated group (6h)	15	14	—	1	—
Baicalin-treated group (12h)	15	15	—	—	—
Octreotide-treated group (3h)	15	8	5	2	—
Octreotide-treated group (6h)	15	11	2	1	1
Octreotide-treated group (12h)	15	15	—	—	—

**Table 8:** Comparison of Bcl-2 protein expression in all groups ($M\left(Q_{R}\right)$).

Groups	3 hours	6 hours	12 hours
Sham operation group	0.00(0.00)	0.00(0.00)	0.00(0.00)
Model group	0.00(0.00)	0.00(1.00)	0.00(0.00)
Baicalin-treated group	0.00(0.00)	0.00(1.00)	0.00(0.00)
Octreotide-treated group	0.00(0.00)	0.00(1.00)	0.00(0.00)

**Table 9:** Changes of Caspase-3 protein expression in all groups.

Groups	Cases	Pathologic grade			
		−	+	++	+++
Sham operation group (3h)	15	15	—	—	—
Sham operation group (6h)	15	15	—	—	—
Sham operation group (12h)	15	15	—	—	—
Model group (3h)	15	13	2	—	—
Model group (6h)	13	11	2	—	—
Model group (12h)	10	8	2	—	—
Baicalin-treated group (3h)	15	13	2	—	—
Baicalin-treated group (6h)	15	10	5	—	—
Baicalin-treated group (12h)	15	10	5	—	—
Octreotide-treated group (3h)	15	14	1	—	—
Octreotide-treated group (6h)	15	13	2	—	—
Octreotide-treated group (12h)	15	12	3	—	—

**Table 10:** Comparison of Caspase-3 protein expression in all groups ($M\left(Q_{R}\right)$).

Groups	3 hours	6 hours	12 hours
Sham operation group	0.00(0.00)	0.00(0.00)	0.00(0.00)
Model group	0.00(0.00)	0.00(0.00)	0.00(0.00)
Baicalin-treated group	0.00(0.00)	0.00(1.00)	0.00(1.00)
Octreotide-treated group	0.00(0.00)	0.00(0.00)	0.00(0.00)
